# Semen Cryopreservation in Testicular Cancer: Before or After Orchidectomy?

**DOI:** 10.1111/andr.70190

**Published:** 2026-02-09

**Authors:** Alessandra Buonacquisto, Carlotta Pozza, Gaia Cicolani, Anna Chiara Conflitti, Luisa Caponecchia, Serena Bianchini, Enrico Delli Paoli, Marta Tenuta, Daniele Gianfrilli, Andrea M. Isidori, Francesco Lombardo, Francesco Pallotti, Donatella Paoli

**Affiliations:** ^1^ Laboratory of Seminology “Loredana Gandini” Sperm Bank Department of Experimental Medicine “Sapienza” University of Rome Rome Italy; ^2^ Department of Medicine and Surgery University of Enna “Kore” Enna Italy; ^3^ Division of Endocrinology and Andrology Department of Experimental Medicine Sapienza University Rome Italy

**Keywords:** ART, hormones, orchidectomy, sperm cryopreservation, sperm DNA fragmentation, testicular cancer

## Abstract

**Background:**

Fertility preservation in patients with testicular cancer remains a clinical priority, yet the optimal timing for sperm cryopreservation—before or after orchidectomy—remains a matter of debate.

**Objectives:**

The aim of this study was to determine the optimal timing for semen cryopreservation and the best‐quality sample for ART. We evaluated various markers, including semen analysis, hormonal profiles, ultrasound analysis and sperm DNA fragmentation (SDF) before and after orchidectomy.

**Material and Methods:**

Comprehensive evaluations were conducted, including semen analysis, hormonal profiling, testicular ultrasound, and SDF assessment.

**Results:**

Post‐orchidectomy samples exhibited a significant decline in total sperm count and progressive motility, as well as an increase in morphological abnormalities. However, a notable and significant reduction in SDF was observed after surgery, suggesting improved chromatin integrity once the tumour had been removed. Elevated preoperative follicle‐stimulating hormone (FSH) and higher body mass index (BMI) were identified as risk factors for post‐operative oligozoospermia. Hormonal assessment revealed increased levels of FSH and luteinising hormone (LH) post‐surgery, with a slight decrease in total testosterone. A significant relationship emerged between testicular volume and changes in SDF: patients with larger contralateral testicular volume experienced a more pronounced improvement in DNA fragmentation, while testicular echotextural heterogeneity was associated with a diminished benefit.

**Discussion and Conclusion:**

Although not all participants underwent full diagnostic evaluation, limiting certain analyses, the findings nonetheless underscore the importance of a detailed andrological assessment at the time of diagnosis. This should include semen analysis, hormonal evaluation and ultrasound examination of the contralateral testis to inform personalised fertility preservation strategies. Despite the deterioration in conventional semen parameters, the post‐orchidectomy sample demonstrated better DNA integrity and may thus represent the more suitable sample for use in assisted reproductive technologies.

## 1 Introduction

Testicular cancer is relatively uncommon, accounting for less than 1% of all male cancers. However, it is the most common solid tumour in men aged 20–34 years and its global incidence has been steadily rising over the past several decades [1]. Most patients with testicular cancer have a primary tumour in the testis, while in less than 5% of cases, the primary tumour is located extragonadally in the retroperitoneum or mediastinum [2]. Of all primary testicular tumours, 95% are germ cell neoplasia in situ (GCNIS) [2, 3]. According to the most recent WHO classification of testicular cancers, these malignancies can be divided into three categories: (1) germ cell tumours derived from GCNIS (GCNIS‐related), (2) germ cell tumours unrelated to GCNIS (GCNIS‐unrelated) and (3) sex cord‐stromal tumours [4]. With cure rates estimated at 90% and a 5‐year survival rate greater than 95%, testicular cancer is one of the most curable malignancies [5]. The first‐line treatment for all primary tumours is unilateral radical inguinal orchidectomy. This procedure is essential for histopathological and staging assessment, local control of the tumour and potentially curing patients whose disease is confined to the testis [6]. Histologic examination is a critical step in defining the treatment (adjuvant chemotherapy). After correctly staging the patient, all guidelines recommend evaluating, in collaboration with the patient, a personalised treatment approach, considering various treatment options and the different risk profiles of side effects resulting from the chosen therapy. In later stages of the disease, orchidectomy may be followed by radio‐ and/or chemotherapy, which can have long‐term effects such as metabolic syndrome, secondary cancers and infertility [7]. These treatments can significantly impact semen quality and sperm chromatin integrity, as the high cell turnover rate of the seminiferous epithelium makes it highly sensitive to these therapies. This could result in azoospermia and sperm chromatin alterations, which may adversely affect reproductive outcomes [8, 9]. Considering these gonadotoxic effects, the only way to protect the patient's future fertility and provide a potential reproductive opportunity is through cryopreservation of undamaged semen or testicular tissue. Cryopreservation involves storing spermatozoa at −196°C in liquid nitrogen, which arrests metabolic processes and maintains the male gametes in a suspended state until they are used in assisted reproductive technology (ART) [10]. This treatment allows spermatozoa to remain viable indefinitely [10]. According to literature data, sperm cryopreserved for over 10 years retains clinical effectiveness. No significant differences in pregnancy, abortion or live birth rates were observed between short‐term and long‐term (up to 15 years) cryopreservation [11]. Live births have been reported from IUI with semen stored for 28 years and from ICSI with semen stored for 40 years [12, 13]. Cryopreserved sperm is associated with a slightly lower pregnancy and live birth rate than fresh sperm, but the observed differences are unlikely to be clinically meaningful [14]. The main scientific societies, including the American Society for Reproductive Medicine (ASRM), the American Society of Clinical Oncology (ASCO) and the Associazione Italiana di Oncologia Medica (AIOM), strongly recommend semen cryopreservation before starting antineoplastic treatment. In particular, the AIOM Guidelines 2024 suggest cryopreservation before orchidectomy if there is a suspicion of impaired function in the contralateral testis. Practices regarding sperm cryopreservation in testicular cancer patients vary across Europe. The timing of cryopreservation, either before or after orchidectomy, remains a matter of debate and each country tends to follow its own national guidelines In France and the Netherlands, preoperative cryopreservation is commonly proposed, following national guidelines and facilitated by easier access to fertility centres. In contrast, Belgian and Luxembourgish urologists more often suggest cryopreservation after orchidectomy, mainly because of the urgency of surgery [15]. However, the presence of the tumour itself may interfere with spermatogenesis and affect sperm DNA integrity [8, 16, 17]. Current data do not fully agree on the effect of orchidectomy on semen quality. While some studies show that surgery negatively affects semen characteristics, other research suggests that removing the affected testis may improve semen quality by eliminating the paracrine effect of inflammatory factors [18, 19, 20]. These data leave some uncertainty about the optimal timing for offering fertility preservation options to the patient. Differences in spermatogenesis, sperm chromatin integrity and hormonal profiles before and after orchidectomy may offer valuable insights, helping select the best semen sample for ART and guide cryopreservation in patients with suspected testicular neoplasia. In comparison with the general population, testicular cancer survivors had a 33% lower probability of having a child within 5 years after diagnosis and a 20% lower probability after more than 5 years [21]. Data from Norwegian registers showed a reduced probability of fatherhood and greater use of ART in testicular cancer survivors than in the control population [22]. In light of these considerations, the aim of this study was to evaluate various markers, including semen analysis, hormonal profiles, ultrasound analysis and sperm DNA fragmentation (SDF) before and after orchidectomy, to determine the optimal timing for semen cryopreservation and the best‐quality sample for ART.

## 2 Materials and Methods

### 2.1 Patients

The study was designed as a prospective and monocentric study. Patients who received a diagnosis of testicular germ cell tumour (seminoma and non‐seminoma) were recruited. All patients performed semen cryopreservation before and after orchidectomy (within 1 month) at the Laboratory of Seminology–Sperm Bank ‘*Loredana Gandini*’, Department of Experimental Medicine, ‘Sapienza’ University of Rome. Semen analysis, hormonal evaluation, testicular ultrasound and SDF testing were performed. All procedures were performed before and after orchidectomy and before chemo/radiotherapy treatments. The study population included patients with suspected testicular neoplasia, aged over 18 years, who provided written informed consent. The exclusion criteria were:

1. the presence of andrological pathologies (cryptorchidism, clinically relevant varicocoele, hypogonadism, etc.) and/or endocrinological diseases which may interfere with semen quality;
2. azoospermia (both obstructive and non‐obstructive);
3. bilateral synchronous tumours;
4. the presence of an abnormal karyotype and other known genetic conditions;
5. previous treatment(s) with chemo/radiotherapy and/or potentially gonadotoxic drugs for oncological pathologies;
6. previous treatment(s) with potentially gonadotoxic drugs for non‐oncological pathologies.

### 2.2 Semen Analysis and Sperm Cryopreservation

Semen samples were collected by masturbation after a minimum of 2 days and a maximum of 7 days of ejaculatory abstinence. All samples were allowed to liquefy at 37°C for 60 min and were then assessed according to WHO [23]. The following variables were taken into consideration: volume (mL), total sperm number (*n* × 10^6^ per ejaculate), progressive motility (%) and morphology (% abnormal forms). For cryopreservation after semen analysis, each sample was diluted (1:1) with freezing medium (Freezing Medium Test Yolk Buffer; Irvine Scientific, Santa Ana, CA, USA). After equilibration for 15 min at 37°C, the mixture was aspirated into 0.50 mL straws and powder sealed. The straws were frozen in liquid nitrogen vapour for 8 min and then stored in liquid nitrogen at −196°C.

### 2.3 Hormone Evaluation

Recruited subjects provided a peripheral blood sample at around 8 AM after an overnight fast. It was performed both before and after orchidectomy. Follicle‐stimulating hormone (FSH), luteinising hormone (LH) and total testosterone (TT) were quantified by Chemiluminescent Microparticle Immunoassay (CMIA, Architect System; Abbott Laboratories, Abbott Park, IL, USA). The detection limits, intra‐ and inter‐assay coefficients of variation and normal ranges were previously described by Pallotti et al. [24].

### 2.4 Ultrasound Analysis

Testicular ultrasounds (US) were all performed with the same machine using a Philips IU22 unit (Philips, Bothell, WA, USA) with a 5–17 MHz wideband linear transducer. All images were stored on a server and reviewed by an experienced second‐party operator, who was not involved in the original examination and was unaware of the patients’ clinical data. All US examinations included the evaluation of the following parameters: testicular volume (TV), echotexture, echogenicity, presence of a varicocoele and the presence of testicular microlithiasis (TML) [25, 26]. TV was evaluated using the ellipsoid formula (height × width × length [cm] × 0.523) and expressed in millilitres. TV was considered reduced if it was below 12 mL [26]. Overall testicular echotexture was categorised as either homogeneous or inhomogeneous, and echogenicity was graded as normal or reduced. Varicocoele and TML were classified in accordance with the European Academy of Andrology (EAA) classification [26]. Both conditions were analysed as dichotomous variables (absent/present), in line with the study design. The data analysed pertain to the contralateral testis, unaffected by testicular cancer.

### 2.5 Sperm DNA Fragmentation (SDF)

SDF was evaluated using the TUNEL assay (Roche, In Situ Cell Death Detection Kit, Fluorescein, Roche, Basel, Switzerland). An aliquot of samples was centrifuged and processed as previously described by Gandini et al. [27]. The samples were then analysed under a fluorescence microscope (Leica DMR; Leica, Wetzlar, Germany), counting at least 500 cells.

### 2.6 Statistical Analysis

Outcome measurements were assessed for normality using the Shapiro–Wilk test, and non‐parametric tests were used when violations of parametric test assumptions were evident. Values are expressed as the median and interquartile range (IQR) for continuous variables. Comparisons between pre‐ and post‐orchidectomy values were performed using the Wilcoxon signed‐rank test for paired samples. Independent groups were compared using the Mann–Whitney U test for continuous variables and the Chi‐square test or Fisher's exact test for categorical variables, as appropriate. An appropriately corrected linear regression model, using bootstrap regression analysis (1000 iterations) to provide the most robust estimates, was performed to evaluate which US or hormonal characteristics, significant in univariate analysis, could be potential predictors of changes in pre‐ and post‐orchidectomy sperm characteristics. Multivariable logistic regression models were applied to assess predictors of dichotomous post‐orchidectomy semen outcomes with covariates selected a priori based on clinical relevance. As the analyses were hypothesis‐driven and did not involve multiple pairwise group comparisons, no post hoc tests or *p* value adjustments were applied. The *p* values were two‐sided, and a threshold of *p* < 0.05 was considered statistically significant. All statistical analyses were conducted using SPSS Statistics version 27.0 (IBM SPSS Statistics Inc., Chicago, IL, USA).

## 3 Results

A total of 176 patients (114 seminomas and 62 non‐seminomas) were recruited for the study. Semen analysis was performed in all 176 patients, hormonal evaluation in 160 patients, testicular ultrasound in 114 patients and SDF was evaluated in 48 patients.

### 3.1 Semen Parameters

**FIGURE 1:**
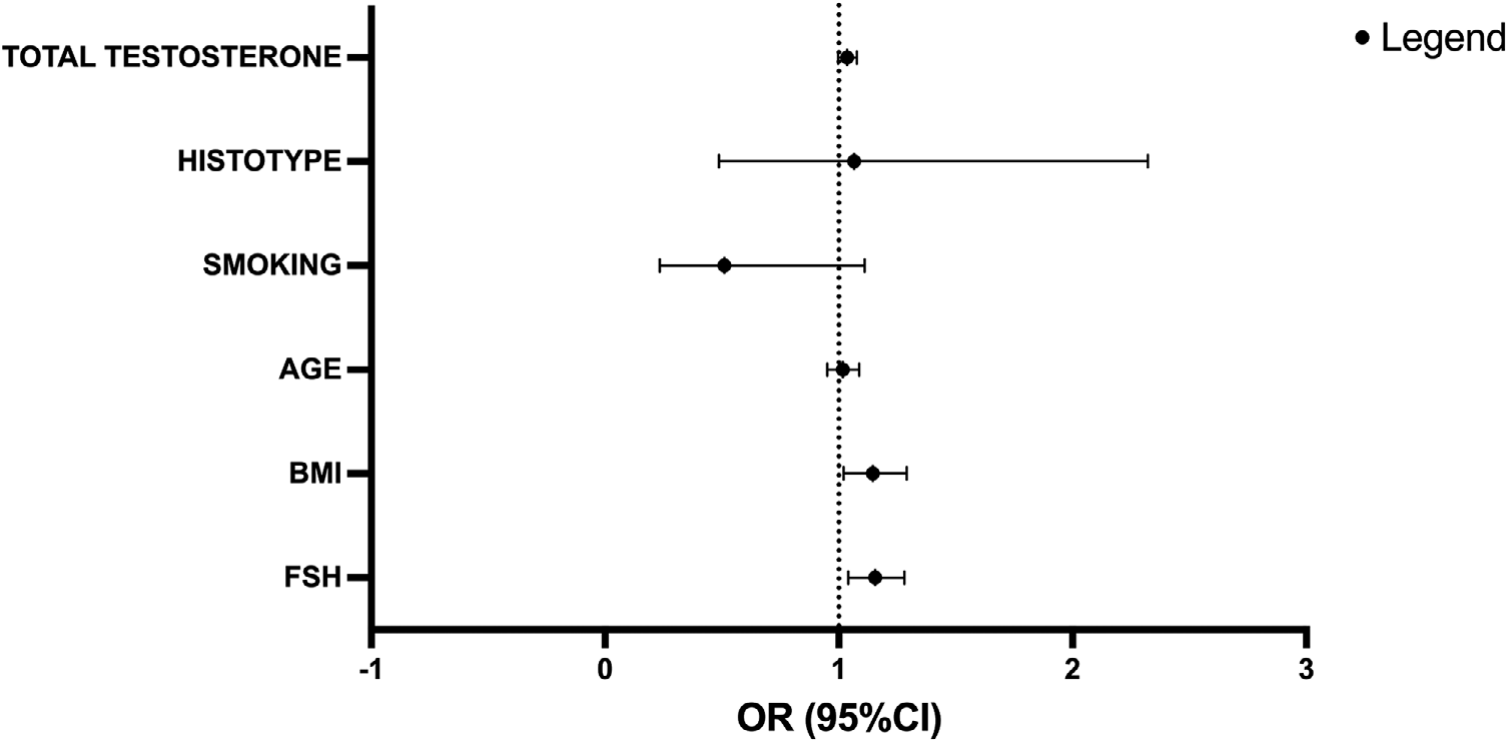
Forest plot illustrating the association between pre‐operative variables and the risk of post‐orchidectomy oligozoospermia. Points represent odds ratios (ORs), and horizontal bars indicate 95% confidence intervals (CIs).

Semen analysis was performed for 176 patients both before and after orchiectomy, but prior to any adjuvant treatment. The median age of patients before orchiectomy was 31.0 years, and median body mass index (BMI) was 24.2 kg/m^2^. Current smokers were 57 (33%). Table 1 describes semen parameters before and after surgery: a decrease in total sperm count and progressive motility, and an increase in the percentage of abnormal forms were observed. A logistic regression model to evaluate the likelihood of post‐orchidectomy oligozoospermia based on pre‐operative variables was performed. We found that higher pre‐orchidectomy FSH levels and higher BMI were significantly associated with increased odds of post‐orchidectomy oligozoospermia. Specifically, for each 1‐unit increase in FSH, the odds of developing oligozoospermia increase by 15.5%, and for each 1‐unit increase in BMI, the odds of developing oligozoospermia increase by 14.5% (Table 2 and Figure 1). In the evaluation of other post‐orchidectomy semen endpoints, after adjustment for age, BMI, histotype, pre‐operative FSH and TT, FSH and BMI were independently associated with progressive asthenozoospermia (OR 1.180, *p* = 0.001; OR 1.185, *p* = 0.012), with a concurrent effect of age (OR 1.081, *p* = 0.029) (Table S1). Similarly, FSH and BMI were associated with teratozoospermia (OR 1.110, *p* = 0.006 and OR 1.164, *p* = 0.021, respectively), whereas age was not significant; histotype and TT were not associated in either model (Table S2).

**TABLE 1:** Semen parameters (*n* = 176 patients) and hormonal profile (*n* = 160 patients) before and after orchidectomy.

Parameters	Before orchidectomy	After orchidectomy	*p* value
Age	31.0 (26.0–36.0)	31.0 (26.0–36.0)	
BMI (kg/m^2^)	24.2 (22.6–26.9)	24.2 (22.6–26.9)	
Abstinence (day)	4.0 (3.0–4.0)	4.0 (3.0–4.0)	0.877
Volume (mL)	3.0 (2.2–4.0)	3.0 (2.4–4.0)	0.526
Total number (x10^6^)	75.0 (27.0–166.4)	58.0 (15.0–147.7)	**<0.001**
Progressive motility (%)	45.0 (25.0–50.0)	40.0 (17.5–50.0)	**0.013**
Abnormal form (%)	88.5 (86.0–94.0)	89.2 (87.0–96.0)	**0.020**
Sperm vitality (%)	74.0 (50.7–80.0)	71.0 (45.5–80.0)	0.098
FSH (mUI/mL)	4.3 (2.0–7.3)	7.6 (5.0–12.2)	**<0.001**
LH (mUI/mL)	3.0 (1.6–4.5)	4.4 (3.1–6.1)	**<0.001**
TT (nmol/L)	18.8 (14.5–24.2)	18.0 (14.3–21.9)	**0.013**

*Note*: Data are expressed in median and interquartile range (IQR) for continuous variables. Comparisons between pre‐ and post‐orchidectomy values were performed using the Wilcoxon signed‐rank test for paired samples. Significant values are indicated in bold.

Abbreviations: FSH, follicle‐stimulating hormone; LH, luteinising hormone; TT, total testosterone.

**TABLE 2:** Logistic regression model assessing predictors of post‐orchidectomy oligozoospermia.

Independent variable	B	SE	OR	95% CI	*p* value
Age	0.016	0.035	1.017	0.950–1.088	0.634
BMI	0.136	0.061	1.145	1.016–1.291	**0.027**
Smoking	−0.674	0.397	0.510	0.234–1.110	0.090
Histotype	0.063	0.398	1.065	0.488–2.322	0.874
FSH (before orchidectomy)	0.144	0.045	1.155	1.058–1.260	**<0.001**
TT (before orchidectomy)	0.035	0.020	1.036	0.997–1.077	0.073

*Note*: Dependent variable: Presence of oligozoospermia after orchidectomy (yes/no). Significant values are indicated in bold.

Abbreviations: BMI, body mass index; FSH, follicle‐stimulating hormone; Histotype, seminoma, non‐seminoma; TT, total testosterone.

### 3.2 Hormone Levels

Concentrations of FSH, LH and TT were evaluated for 160 patients before and after orchidectomy. Table 1 describes hormone concentrations before and after surgery. As expected, an increase in FSH and LH was observed after orchidectomy (*p* < 0.001), while TT showed a slight reduction at the two time points (*p* = 0.013).

### 3.3 Sperm DNA Fragmentation

SDF analysis was performed for 48 patients (Table 3). We observed a decrease in SDF after orchidectomy (*p* < 0.001). Using linear models, we did not identify the presence of predictor variables among clinical characteristics before orchidectomy for variation in DNA fragmentation (Table 4).

**TABLE 3:** Sperm DNA fragmentation (SDF) before and after orchidectomy.

	Before orchidectomy, *N* = 48	After orchidectomy, *N* = 48	*p* value
**SDF (%)**	14.5 (9.3–24.1)	10.2 (6.0–17.2)	**<0.001**

*Note*: Data are expressed as median and interquartile range (IQR). Comparisons between pre‐ and post‐orchidectomy values were performed using the Wilcoxon signed‐rank test for paired samples. Signficant values are indicated in bold.

Abbreviation: SDF, sperm DNA fragmentation.

**TABLE 4:** Linear regression model evaluating predictors of change in sperm DNA fragmentation (ΔSDF).

	B	SE	Beta	*p* value	95% CI	
Age	−0.097	0.185	−0.106	0.602	−0.473	0.278
BMI	0.146	0.377	0.073	0.700	−0.620	0.913
Histotypes	−4.109	2.200	−0.335	0.070	−8.580	0.363
Total sperm number (x10^6^, before orchidectomy)	0.014	0.009	0.289	0.106	−0.003	0.032
FSH (mUI/mL, before orchidectomy)	0.354	0.265	0.252	0.190	−0.184	0.892
TT (nmol/L, before orchidectomy)	0.014	0.104	0.025	0.893	−0.197	0.225

*Note*: The overall model was not statistically significant (F (6,34) = 1.091, *p* = 0.387), with an *R* ^2^ = 0.161 and an adjusted *R* ^2^ = 0.014. Dependent variable: ΔSDF% (SDF after orchidectomy – SDF before orchidectomy).

Abbreviations: BMI, body mass index; FSH, follicle‐stimulating hormone; Histotypes, seminoma, non‐seminoma; TT, total testosterone.

### 3.4 Ultrasound Analysis

Ultrasound data were available for 114 out of 176 patients, as only those who underwent a pre‐orchidectomy ultrasound at our centre had their imaging parameters recorded. The remaining patients had their ultrasound examinations performed externally, without standardised reporting or access to full imaging details. In the ultrasound subset (*n* = 114), the median contralateral testicular volume was 15.0 mL (IQR 11.5–18.1). A Spearman correlation analysis was performed to assess the relationship between contralateral TV and changes in semen parameters after orchidectomy. TV was significantly correlated with sperm DNA fragmentation changes (ΔSDF) (*r* = 0.402, *p* = 0.015), indicating that a larger TV was associated with greater improvement in sperm DNA integrity. To further assess the predictive value of TV on ΔSDF, a linear regression analysis was performed. TV appeared to be a significant predictor of ΔSDF (*B* = 0.411, *p* = 0.022), meaning that larger contralateral TV predicts a greater reduction in SDF after orchidectomy. Conversely, testicular structural heterogeneity was negatively associated with ΔSDF (*B* = −4.350, *p* = 0.049). Testicular echogenicity (*p* = 0.289) and the presence of microlithiasis (*p* = 0.138) were not significantly associated with ΔSDF (Table 5). On univariable analysis, the presence of varicocoele was not associated with ΔSDF (*B* = −0.111; *p* = 0.965). In the multivariable model, adding varicocoele did not change the effect estimates for TV (*B* = 0.404; 95% CI 0.038–0.770; *p* = 0.032) and varicocoele itself remained non‐significant. Additionally, patients were divided into two groups based on TV (normal TV if > 12 mL, *n* = 83 and low TV if < 12 mL, *n* = 35), to assess whether the pre‐ and post‐orchidectomy differences in semen parameters differed between these two groups. SDF decreased significantly in both groups (13.4% vs. 8.9%, *p* < 0.001 in normal TV and 39.1% vs. 32.0%, *p* = 0.043 in low TV group). FSH and LH levels increased significantly in both groups (*p* < 0.001), and TT decreased only in the low TV group (*p* = 0.009). Total sperm count decreased (*p* = 0.021), as well as progressive motility (*p* = 0.039) in low TV group. Sperm vitality showed a near‐significant decrease (*p* = 0.051) only in low TV group.

**TABLE 5:** Linear regression model assessing the relationship between testicular ultrasound parameters of the contralateral testicle and change in sperm DNA fragmentation (ΔSDF).

	B	SE	Beta	*p* value	95% CI	
TV	0.411	0.168	0.447	**0.022**	0.064	0.758
Testicular echotexture	−4.350	2.090	−0.411	**0.049**	−8.674	−0.027
Testicular echogenicity	3.376	3.111	0.225	0.289	−3.060	9.812
TML	4.140	2.694	0.324	0.138	−1.434	9.713

*Note*: Dependent variable: ΔSDF. The overall model was statistically significant (F (4,23) = 3.310, *p* = 0.028), with an *R* ^2^ = 0.365 and adjusted *R* ^2^ = 0.255. Significant values are indicated in bold.

Abbreviations: ΔSDF: change in sperm DNA fragmentation (post‐orchidectomy – pre‐orchidectomy); SDF: sperm DNA fragmentation; TML, testicular microlithiasis; TV, testicular volume.

## 4 Discussion

Male fertility can be impaired by cancer itself and cancer therapy, including chemotherapy and radiotherapy. The successes of current therapeutic strategies make it possible to focus on improving the long‐term quality of life of patients, especially in terms of fertility and parenthood [28, 29]. From this perspective, sperm banking for male cancer patients is considered the most effective method for preserving fertility. Our centre, the Sperm Bank ‘*Loredana Gandini*’, Policlinico Umberto I, Rome, has performed sperm cryopreservation in patients diagnosed with cancer since 1996. From June 1996 to December 2024, our unit has cryopreserved about 7200 sperm samples. The most frequent type of malignancies for which cryopreservation has been performed is testicular cancer (47%), followed by lymphomas (25%). The other pathological conditions (28%) include sarcoma (3%), brain cancer (2%), prostate cancer (2%), leukaemia (2%), bladder neck obstruction (3%) and others (16%). In the context of testicular cancer, clinicians send patients to our centre both before and after tumour excision. Specifically, our analysis showed that 23% of patients with testicular cancer performed cryopreservation before tumour excision, while 77% did so after tumour excision. Given the high rate of cryopreservation in these patients and the subsequent use of the cryopreserved samples for ART, it is essential to assess the cytological and molecular quality of the samples. In our cohort, the percentage of patients who used their cryopreserved sperm samples was 8.5%. Scientific societies strongly recommend cryopreservation before the start of antineoplastic therapies as a result of their high gonadotoxic effects. However, the literature does not clearly define the appropriate timing for cryopreservation in relation to tumour removal surgery [18, 19, 20].

### 4.1 Semen Parameters

Most literature data show low sperm count and quality in testicular cancer patients, suggesting two possible explanations: (i) some patients may be affected by the testicular dysgenesis syndrome (TDS), which includes altered spermatogenesis; (ii) the presence of the tumour itself may interfere with spermatogenesis, although the mechanisms underlying this alteration are not known [30, 31, 32]. Regarding TDS, the combined effects of environmental and genetic factors may alter Sertoli and Leydig cell function, leading to impaired gonadal function and testicular cancer [33]. Concerning the effect of cancer itself, increased blood flow as a consequence of tumour neovascularisation, intrascrotal temperature elevation and human chorionic gonadotropin (βhCG) production have been proposed [17, 34, 35]. However, there are few studies in the literature that compare semen samples before and after orchidectomy. Petersen et al. evaluated the hormonal profile and semen parameters in patients with testicular cancer before and after orchidectomy. The study included 48 patients, 35 of whom underwent semen analysis and 47 hormonal analyses. Thirty patients showed a significant reduction in semen parameters after surgery, and three patients were azoospermic (9%). Specifically, the authors observed a decrease in sperm concentration (median 17 × 10^6^/mL vs. 7 × 10^6^/mL) and total sperm count (median 39×10^6^ vs. 30 × 10^6^) between before and after orchidectomy samples. The authors concluded that the optimal timing for sperm cryopreservation would be prior to orchidectomy, as semen parameters show a significant decline after the surgery [18]. Similarly, Liguori et al. (2008) conducted an analysis of semen parameters before and after surgery in 30 patients with a mean age of 32.7 years. A significant reduction in sperm concentration was observed (*p* = 0.001), from a mean of 26.7 × 10^6^/mL before surgery to 16.6 × 10^6^/mL after surgery. The 30 patients were divided into two groups based on histological type: one group with 18 patients affected by seminoma, and a second group of 12 patients with non‐seminoma. In the seminoma group (*n* = 18), the mean sperm concentration before and after surgery was 33.5 × 10^6^/mL and 22.6 × 10^6^/mL, respectively (*p* = 0.004). In the non‐seminoma group (*n* = 12), the mean sperm concentration before and after surgery was 16.4 × 10^6^/mL and 7.5 × 10^6^/mL, respectively (*p* = 0.046). Furthermore, Liguori et al. (2008) observed that pre‐orchidectomy sperm concentration was significantly lower in non‐seminomas (16.4 × 10^6^/mL) compared to seminomas (33.5 × 10^6^/mL) (*p* = 0.004). The authors emphasised that following orchidectomy, there is a significant reduction in sperm quality when comparing pre‐ and post‐surgery semen parameters. Patients with testicular cancer already show altered seminal parameters before orchidectomy, especially in non‐seminomas [19]. More recently, Andrade et al. conducted a study on 24 patients with testicular cancer, examining semen quality before and after orchidectomy. Although not statistically significant, after orchidectomy, 50% of patients showed reduced sperm concentration and motility, 54.2% had lower semen volume, and 20.8% showed decreased normal sperm morphology. The authors reported that semen parameters were already impaired before orchidectomy, indicating a tumour‐related impairment of spermatogenesis [20]. In our cohort of patients, a significant decrease in total sperm count and progressive motility and a significant increase in the percentage of abnormal forms was observed. This finding suggests that removing the testis affected by the tumour can cause a deterioration in semen quality.

### 4.2 Hormone Levels

The topic of hormonal levels in patients with testicular neoplasia, both before and after orchidectomy, is of great importance for understanding gonadal function and providing appropriate oncological counselling. Several studies have examined this aspect, although the number of studies is limited. Petersen et al. were among the first to report relevant findings. In a study involving 47 patients, they observed a significant increase in FSH levels from 5.7 IU/L pre‐orchidectomy to 10 IU/L post‐orchidectomy, while LH levels rose from 3.1 IU/L to 5.2 IU/L (*p* < 0.001). Despite the reduction in Leydig cells following orchidectomy, androgen production appears to be maintained owing to the increase in LH. Inhibin B levels dropped from 108 to 95 pg/mL (*p* = 0.003), indicating reduced spermatogenesis, while androgen production seemed unaffected [18]. The study by Tomomasa et al. investigated gonadal function in 18 patients with testicular cancer, assessing both semen parameters and hormonal levels. The results indicated an increase in FSH, LH, and prolactin (PRL) levels following orchidectomy. Regarding TT, its levels increased in four out of six patients, while in two patients, they decreased [36]. Irkilata et al. analysed a cohort of 40 adult patients with cryptorchidism who did not have testicular neoplasia. The results suggest that orchidectomy did not cause significant changes in semen parameters or TT levels, except in patients with histopathological findings of maturational arrest. In these patients, an increase in FSH levels and a significant reduction in inhibin B were observed, likely as a result of the loss of germ cells at early maturational stages. In summary, studies suggest that after orchidectomy, FSH levels tend to increase, LH levels rise slightly, and inhibin B decreases [37]. Wiechno et al. assessed hormonal levels (TT, LH, FSH, estradiol, prolactin) and βhCG in 128 patients with unilateral testicular tumours before orchidectomy, 1 month after orchidectomy and at least 1 year after treatment. TT levels were found to be significantly lower after orchidectomy, with 16% of patients showing TT levels below 2.31 ng/mL, 1 month after surgery. This number increased to 17%, 1‐year post‐treatment. Both LH and FSH levels increased significantly after orchidectomy, reflecting a disruption in the normal feedback regulation as a consequence of testicular damage. This trend persisted at the 1‐year follow‐up [38]. According to these data, in our cohort study we found a statistically significant increase in FSH and LH after orchidectomy, while TT showed a slightly reduction at the two time points (*p* = 0.013). However, the values largely remained within normal reference ranges following orchidectomy. These results could suggest that despite the reduction in Leydig cells, androgen production is partially maintained because of the rise in LH concentrations. We also found that higher pre‐orchidectomy FSH and BMI were significantly associated with a higher risk of oligozoospermia after orchidectomy. For every 1 unit increase in FSH, the odds of developing oligozoospermia increased by 15.5%, and for every 1 unit increase in BMI, the odds of developing oligozoospermia increased by 14.5%. The same was observed for other semen endpoints, namely impaired progressive motility and teratozoospermia at follow‐up. This important finding suggests that pre‐orchidectomy FSH and BMI may be predictive markers of semen quality after surgery and highlight the importance of a comprehensive andrological evaluation at the time of cancer diagnosis.

### 4.3 Sperm DNA Fragmentation

The integrity of sperm DNA is a crucial characteristic for the accurate transmission of genetic information to the embryo. For this reason, evaluating the integrity of sperm DNA can provide valuable information for determining the molecular quality of a semen sample. SDF has gained importance as a potential marker and the WHO considered this test as an extended examination [23]. A recent review highlighted that the integrity of sperm chromatin in patients with testicular cancer was compromised even before any antineoplastic treatment, although the available data refer to semen samples from patients post‐orchidectomy and pre‐chemotherapy [8]. This could be caused by a maturation defect during spermatogenesis in the remaining testis after orchidectomy, but also by the impact of stress factors, oestrogens and βhCG production, or other factors related to the development of testicular dysplasia [39]. Testicular cancer can indeed be hormonally active, producing βhCG and alpha‐fetoprotein (AFP), and have both local and systemic effects, including temperature increase and metabolic effects [40]. Oncogenesis can, therefore, cause a systemic inflammatory state, and the cytokines secreted can influence the hypothalamic–pituitary–gonadal axis [41]. All of this could also affect sperm molecular quality which could have a negative impact on reproduction. In fact, sperm DNA damage can compromise embryo development, implantation and pregnancy in both natural and assisted reproduction. Literature data show a correlation between SDF and poor embryo quality and slower cleavage rate [9]. For these reasons, the assessment of SDF could be informative during the infertile couple's work‐up to improve the counselling of couples undergoing ART. Andrade et al. studied 24 testicular cancer patients, analysing semen quality, chromatin integrity (Comet Assay), and mitochondrial activity before and after orchidectomy. They observed reduced DNA fragmentation and increased mitochondrial activity after surgery. The authors associated the improvement in chromatin integrity with the increase in mitochondrial activity observed in post‐intervention samples. According to this hypothesis, inactive mitochondria release pro‐oxidative molecules that induce DNA fragmentation, while higher mitochondrial activity reduces oxidative stress [20]. Likewise, Belardin et al. studied apoptotic pathways by comparing the levels of certain proteins in seminal plasma in patients with testicular neoplasia, before and after orchidectomy. In post‐orchidectomy patients, they observed that the Bad protein in seminal plasma was significantly lower, while levels of Bcl2, Akt, Caspase‐9, p53 and Caspase 8 were statistically higher compared to pre‐orchidectomy patients. The authors indicated that testicular neoplasia causes an increase in DNA fragmentation and the alteration of the seminal plasma proteins involved in apoptosis. After orchidectomy, apoptotic pathway proteins are restored, suggesting recovery of apoptotic activity in the contralateral testicle and explaining the decrease in SDF [42]. Consistent with these studies, we observed a significant decrease in SDF after orchidectomy, suggesting tumour‐related DNA damage. We can hypothesise that the inflammatory process caused by the tumour may induce spermatogenic arrest, with the formation of immature forms with less compact chromatin and, thus more sensitive to damage from oxidative stress. It is also possible that the tumour induces intratesticular damage to the apoptosis control system, resulting in the release into the ejaculate of damaged cells that should be eliminated during spermatogenesis. Removal of the tumour induces a reduction in DNA fragmentation. Current smoking was associated with a smaller post‐orchidectomy reduction in SDF (trend‐level, *p* = 0.064), consistent with a potential adverse effect of smoking; however, this analysis was limited by the small SDF subset.

### 4.4 Ultrasound Analysis

Scrotal ultrasound, in addition to its role in the diagnosis and characterisation of testicular tumours, is also essential for the assessment of the contralateral testis [43, 44]. It provides valuable information regarding TV, echogenicity, structural homogeneity and the presence of microlithiasis, which are essential parameters for assessing the functional reserve of the remaining testis and predicting potential changes in semen quality after orchidectomy. A thorough ultrasound examination of the contralateral testis plays a crucial role in assessing the risk of bilateral testicular cancer, as certain ultrasonographic features, including microlithiasis, reduced volume and altered echotexture, have been associated with an increased likelihood of metachronous testicular tumours [45]. In our analysis, contralateral testis volume significantly predicted post‐orchidectomy SDF improvement, suggesting that a larger volume may help maintain spermatogenesis after unilateral testicular loss. In contrast, structural heterogeneity was linked to poorer sperm DNA recovery, indicating that an inhomogeneous parenchyma may reflect underlying spermatogenic dysfunction. The presence of varicocoele was not associated with ΔSDF and did not change the independent association between contralateral TV and SDF improvement, while echotexture lost significance once varicocoele was entered, a finding that may reflect overlapping information between mild varicocoele and parenchymal inhomogeneity in a small analytic subset, since moderate–severe varicocoeles were excluded by protocol. Echogenicity and microlithiasis did not significantly impact sperm DNA fragmentation changes, possibly as a consequence of the limited sample size, dichotomization of variables, or the multifactorial nature of post‐orchidectomy reproductive function, which may not be entirely explained by standard ultrasound parameters alone. These findings underscore the importance of a comprehensive preoperative ultrasound assessment. Assessing the status of the contralateral testis may help in predicting post‐orchidectomy reproductive outcomes and guide fertility counselling. The choice of semen cryopreservation timing should consider not only pre‐orchidectomy semen parameters but also ultrasound‐derived testicular health indicators. Patients with a contralateral testis with low TV are at greater risk of experiencing a decline in sperm count and motility. However, SDF improves regardless of TV, with a greater improvement in patients with normal volume testes. A limitation must be acknowledged: the sample size for sperm DNA fragmentation analyses was reduced as a result of the limited availability of surplus aliquots.

## 5 Conclusion

Male fertility in patients with testicular cancer is affected by both the tumour and its treatment. Pre‐orchidectomy cryopreservation could be useful if the contralateral testis is impaired. Although semen parameters are lower than before surgery, samples from post‐orchidectomy cryopreservation show a better molecular profile. Fertility preservation should be based on a personalised, evidence‐based approach that integrates conventional semen parameters, sperm DNA integrity, and contralateral testicular health when counselling patients on the optimal timing of cryopreservation.

In conclusion, the key points for timing sperm cryopreservation in testicular cancer patients are:

*Before orchidectomy*

1. Higher sperm concentration, motility and normal morphology.
2. Provides the best chance of banking motile spermatozoa.

*After orchidectomy*

1. Semen parameters (count, motility and morphology) tend to decline.
2. Hormonal changes: increase in FSH and LH, decrease in inhibin B, with testosterone generally maintained.
3. Sperm DNA fragmentation improves, suggesting better molecular quality of post‐orchidectomy samples.

*Predictive factors*

1. Larger contralateral testis volume is associated with greater post‐orchidectomy SDF improvement.
2. Structural heterogeneity or reduced volume of the contralateral testis may indicate impaired spermatogenic reserve.
3. Elevated pre‐orchidectomy FSH and higher BMI predict poorer semen quality after surgery.

## Author Contributions

*Study design*: Donatella Paoli, Francesco Pallotti, Alessandra Buonacquisto. *Article drafting*: Alessandra Buonacquisto. *Andrological data collection*: Donatella Paoli, Enrico Delli Paoli. *Molecular assessment*: Alessandra Buonacquisto, Anna Chiara Conflitti, Gaia Cicolani, Luisa Caponecchia, Serena Bianchini. *Ultrasound data collection*: Marta Tenuta, Carlotta Pozza, Daniele Gianfrilli, Andrea M. Isidori. *Statistical analysis*: Francesco Pallotti, Carlotta Pozza. *Data interpretation*: Donatella Paoli, Francesco Pallotti, Francesco Lombardo. *Supervision*: Donatella Paoli, Francesco Lombardo, Francesco Pallotti, Carlotta Pozza, Andrea M. Isidori. All authors revised the manuscript critically.

## Funding

This work was supported by the Italian Ministry of Education and Research (MIUR‐PRIN 2022EHN49M_003) and the “Sapienza” University of Rome—Faculty of Medicine.

## Conflicts of Interest

The authors declare they have no conflicts of interest.

## Ethics Statement

The study was approved by the Ethics Committee of Policlinico Umberto 1 (Prot.0860/2023). Written informed consent was obtained from all study participants.

## Supporting information




**Table 1s**: Logistic regression model assessing predictors of post‐orchiectomy impaired progressive motility BMI: Body mass index; Histotype: Seminoma, Non‐Seminoma; FSH: Follicle‐stimulating hormone; TT: Total testosterone. Dependent variable: Presence of impaired progressive motility after orchiectomy (yes/no).

**Table 2s**: Logistic regression model assessing predictors of post‐orchiectomy teratozoospermia BMI: Body mass index; Histotype: Seminoma, Non‐Seminoma; FSH: Follicle‐stimulating hormone; TT: Total testosterone. Dependent variable: Presence of teratozoospermia after orchiectomy (yes/no).

## Data Availability

The data that support the findings of this study are available from the corresponding author upon reasonable request.
