# Supplementary material for: Semen Cryopreservation in Testicular Cancer: Before or After Orchidectomy?
Source: Andrology. 2026 Feb 9;14(5):1272–81. doi: 10.1111/andr.70190 (PMC13266445; doi:10.1111/andr.70190)
Supplement: Supplementary file 1 — Table 1s: Logistic regression model assessing predictors of post‐orchiectomy impaired progressive motility BMI: Body mass index; Histotype: Seminoma, Non‐Seminoma; FSH: Follicle‐stimulating hormone; TT: Total testosterone. Dependent variable: Presence of impaired progressive motility after orchiectomy (yes/no). [file ANDR-14-1272-s001.docx]

| Independent variable | B | SE | OR | 95% CI | p-value |
| --- | --- | --- | --- | --- | --- |
| Age | 0.078 | 0.036 | 1.081 | 1.008-1.159 | **0.029** |
| BMI | 0.170 | 0.068 | 1.185 | 1.038-1.353 | **0.012** |
| Histotype | -0.105 | 0.420 | 0.901 | 0.395-2.053 | 0.803 |
| FSH (before Orchiectomy) | 0.166 | 0.048 | 1.180 | 1.074-1.297 | **<0.001** |
| TT (before Orchiectomy) | 0.023 | 0.021 | 1.023 | 0.982-1.066 | 0.269 |

**Table 1s**. **Logistic regression model assessing predictors of post-orchiectomy impaired progressive motility** BMI: Body mass index; Histotype: Seminoma, Non-Seminoma; FSH: Follicle-stimulating hormone; TT: Total testosterone. Dependent variable: Presence of impaired progressive motility after orchiectomy (yes/no).
