# Supplementary material for: Semen Cryopreservation in Testicular Cancer: Before or After Orchidectomy?
Source: Andrology. 2026 Feb 9;14(5):1272–81. doi: 10.1111/andr.70190 (PMC13266445; doi:10.1111/andr.70190)
Supplement: Supplementary file 2 — Table 2s: Logistic regression model assessing predictors of post‐orchiectomy teratozoospermia BMI: Body mass index; Histotype: Seminoma, Non‐Seminoma; FSH: Follicle‐stimulating hormone; TT: Total testosterone. Dependent variable: Presence of teratozoospermia after orchiectomy (yes/no). [file ANDR-14-1272-s002.docx]

| Independent variable | B | SE | OR | 95% CI | p-value |
| --- | --- | --- | --- | --- | --- |
| Age | -0.10 | 0.036 | 0.990 | 0.922-1.063 | 0.775 |
| BMI | 0.152 | 0.066 | 1.164 | 1.023-1.324 | **0.021** |
| Histotype | -0.193 | 0.427 | 0.825 | 0.357-1.904 | 0.652 |
| FSH (before Orchiectomy) | 0.104 | 0.038 | 1.110 | 1.030-1.196 | **0.006** |
| TT (before Orchiectomy) | -0.001 | 0.023 | 0.999 | 0.956-1.045 | 0.980 |

**Table 2s**. **Logistic regression model assessing predictors of post-orchiectomy teratozoospermia** BMI: Body mass index; Histotype: Seminoma, Non-Seminoma; FSH: Follicle-stimulating hormone; TT: Total testosterone. Dependent variable: Presence of teratozoospermia after orchiectomy (yes/no).
